# Atheroprotective role of vinpocetine: an old drug with new indication

**DOI:** 10.1007/s10787-024-01529-5

**Published:** 2024-08-14

**Authors:** Mohammed H. Abu-Alghayth, Hayder M. Al-kuraishy, Ali I. Al-Gareeb, Athanasios Alexiou, Marios Papadakis, Mostafa M. Bahaa, Mohammed Afifi, Ammar AL-Farga, Eman Wahsh, Gaber El-Saber Batiha

**Affiliations:** 1 Department of Medical Laboratory Sciences, College of Applied Medical Sciences, University of Bisha, P.O. Box 255, 67714 Bisha, Saudi Arabia; 2 Department of Clinical Pharmacology and Medicine, College of Medicine, Mustansiriyah University, Baghdad, Iraq; 3 University Centre for Research and Development, Chandigarh University, Chandigarh-Ludhiana highway, Mohali, Punjab India; 4 Department of Research and Development, Funogen, 11741 Athens, Greece; 5 Department of Research and Development, AFNP Med, 1030 Vienna, Austria; 6 Department of Science and Engineering, Novel Global Community Educational Foundation, Hebersham, NSW 2770 Australia; 7 Department of Surgery II, University Hospital Witten-Herdecke, University of Witten-Herdecke, Heusnerstrasse 40, 42283 Wuppertal, Germany; 8 Pharmacy Practice Department, Faculty of Pharmacy, Horus University, New Damietta, Egypt; 9 Department of Biochemistry, College of Sciences, University of Jeddah, Jeddah, Saudi Arabia; 10 Department of Pharmacology and Toxicology, Faculty of Pharmacy, Sinai University, Arish Campus, Arish, 45511 Egypt; 11 Department of Pharmacology and Therapeutics, Faculty of Veterinary Medicine, Damanhour University, Damanhour, 22511 AlBeheira Egypt

**Keywords:** Atherosclerosis, Endothelial dysfunction, Vinpocetine, Pro-inflammatory cytokines, Reactive oxygen species

## Abstract

Endothelial dysfunction is considered one of the main causes of atherosclerosis and elevated blood pressure. Atherosclerosis (AS) formation is enhanced by different mechanisms including cytokine generation, vascular smooth muscle cell proliferation, and migration. One of the recent treatment toward endothelial dysfunction is vinpocetine (VPN). VPN is an ethyl apovincaminate used in the management of different cerebrovascular disorders and endothelial dysfunction through inhibition of atherosclerosis formation. VPN is a potent inhibitor of phosphodiesterase enzyme 1 (PDE1) as well it has anti-inflammatory and antioxidant effects through inhibition of the expression of nuclear factor kappa B (NF-*κ*B). VPN has been shown to be effective against development and progression of AS. However, the underlying molecular mechanism was not fully clarified. Consequently, objective of the present narrative review was to clarify the mechanistic role of VPN in AS. Most of pro-inflammatory cytokines released from macrophages are inhibited by the action of VPN via NF-*κ*B-dependent mechanism. VPN blocks monocyte adhesion and migration by inhibiting the expression of pro-inflammatory cytokines. As well, VPN is effective in reducing oxidative stress, a cornerstone in the pathogenesis of AS, through inhibition of NF-*κ*B and PDE1. VPN promotes plaque stability and prevent erosion and rupture of atherosclerotic plaque. In conclusion, VPN through mitigation of inflammatory and oxidative stress with plaque stability effects could be effective agent in the management of endothelial dysfunction through inhibition of atherosclerosis mediators.

## Introduction

**Fig. 1:**
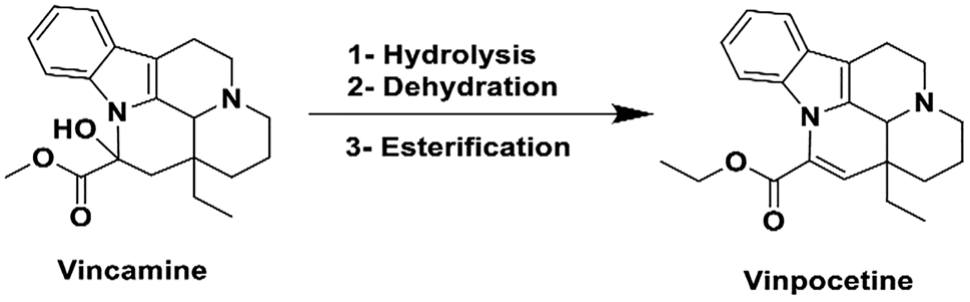
Semi synthesis of vinpocetine from natural alkaloid vincamine

Vinpocetine (VPN) is an ethyl apovincaminate derived from vinca alkaloid vincamine (Fig. 1) which is extracted from *Voacanga africana* seeds and Vinca minor leaves (Ferreira 2021).

VPN was discovered in 1978 in Hungary, and have been broadly used in diverse cerebrovascular disorders (Al-Kuraishy et al. 2020). VPN was first marketed in Hungary as Cavinton by Gedeon Richter Ltd., Budapest, Hungary (formerly Organon), and it is one of their best-selling products in their central nervous system portfolio to this day (Petric et al. 2023). Since the 1980s, the use of vinpocetine has spread to countries including Germany, Poland, Russia, Japan, Portugal, and others, where it was used as a supportive treatment in acute ischemic stroke (Al-Kuraishy et al. 2022a). VPN was used in the management of dementia and stroke in European and Asian countries, though it was not approved for therapeutic use in USA (Al-Kuraishy et al. 2022b). Despite of the extensive uses of VPN as nootropic, it was not approved by FDA. VPN was first used in 1978 in treating dementia, stroke, and memory disorders (Al-kuraishy and Al-Gareeb 2020).

The mechanism of VPN is related to inhibition of sodium channel, reduction of calcium influx and antioxidant effects (Al-Kuraishy, Al-Gareeb, and Al-Nami 2019). VPN is regarded as potent inhibitor of phosphodiesterase enzyme 1 (PDE1). VPN has anti-inflammatory effects by inhibiting the expression of nuclear factor kappa B (NF-*κ*B) through stabilization of I*κ*B which is an inhibitor of NF-*κ*B. Furthermore, VPN has anti-platelet activity by which it can improve cerebral blood flow and brain metabolism (Zhang et al. 2018).

Currently, VPN is also available in the market as a dietary supplement to enhance cognition and memory. Due to its excellent safety profile, increasing efforts have been put into exploring the novel therapeutic effects and mechanism of actions of VPN in various cell types and disease models. Recent studies have revealed a number of novel functions of VPN, including anti-inflammation which antagonize vascular remodeling and atherosclerosis (AS) development. These findings may reposition VPN for preventing or treating relevant disorders in humans (Zhang and Yang 2014). Prolong use of VPN is associated with development of some side effects such as hypotension, tachycardia, dizziness, dry mouth, nausea, heartburn, and flushing (Bönöczk et al. 2000). VPN has specific pharmacokinetic profile; the effective therapeutic dosage of VPN is 5–10 mg (Medina 2010). VPN half-life is 1–2 h; it is highly absorbed from intestine with 56.6% bioavailability, peak plasma level is reached after 1 h of oral administration, highly distributed, cross blood–brain barrier (BBB), metabolized by liver, and excreted by urine (Ping et al. 2019). It has been reported that VPN was effective against development and progression of AS (Zhang and Yang 2014). Though, the underlying molecular mechanism was not fully clarified. Consequently, objective of the present narrative review was to clarify the mechanistic role of VPN in AS.

## Atherosclerosis overview

AS is a disease of medium- and large-size arteries characterized by fatty deposition in the inner part, peripheral thickening of arterial wall, and formation of atherosclerotic plaques (Libby 2021). AS impedes blood flow causing tissue ischemia mostly in the brain and heart leading to stroke and ischemic heart disease correspondingly (Fok and Lanzer 2018). AS complications such as peripheral vascular disease, stroke, and myocardial infarction are the major causes of mortality. Of note, AS process may be started in childhood and become clinically evident in the middle age and later (Schipper and de Ferranti 2022). Rupture of atherosclerotic plaques and associated thromboembolic disorders are the main reasons for cardiovascular complications (Vergallo and Crea 2020). The underlying associated pathological conditions connected with AS progression are inflammation, oxidative stress, endothelial dysfunction, apoptosis, vascular proliferation, matrix degeneration, and neovascularization (Shi et al. 2020).

Hypercholesterolemia is considered as the chief inducer of AS, as increasing of circulating cholesterol increases endothelial permeability, promoting deposition of lipid particles in the vascular endothelium (Rasheed et al. 2021; Al-Maiahy et al. 2021). Lipid particles mostly oxidized LDL (ox-LDL) in the sub-endothelial space act as chemo-attractants for the monocytes which is converted to foamy macrophages. Furthermore, ox-LDL in the sub-endothelial space activates the expression of scavenger receptors on the macrophages with further accumulation of cholesterol in sub-endothelial space. These pathological changes trigger plaque formation, narrowing of vascular lumen and development of AS (Khatana et al. 2020). Atherosclerotic plaques are vulnerable for erosion, rupture, and calcification with nodule formation, higher infiltration of T cells into atherosclerotic plaque increases vulnerability for rupture and thrombosis (Chiorescu et al. 2022).

High LDL and TG with low HDL act as strong predictors for the development of premature AS (Kadhim et al. 2019). However, high HDL level is considered as a protective factor against the development and progression of AS. Moreover, hypertriglyceridemia is regarded as independent risk factor for progression of AS. Similarly, lipoprotein disorders are associated with AS pathophysiology, increased lipoprotein A is related with AS development (Gill et al. 2021).

**Fig. 2:**
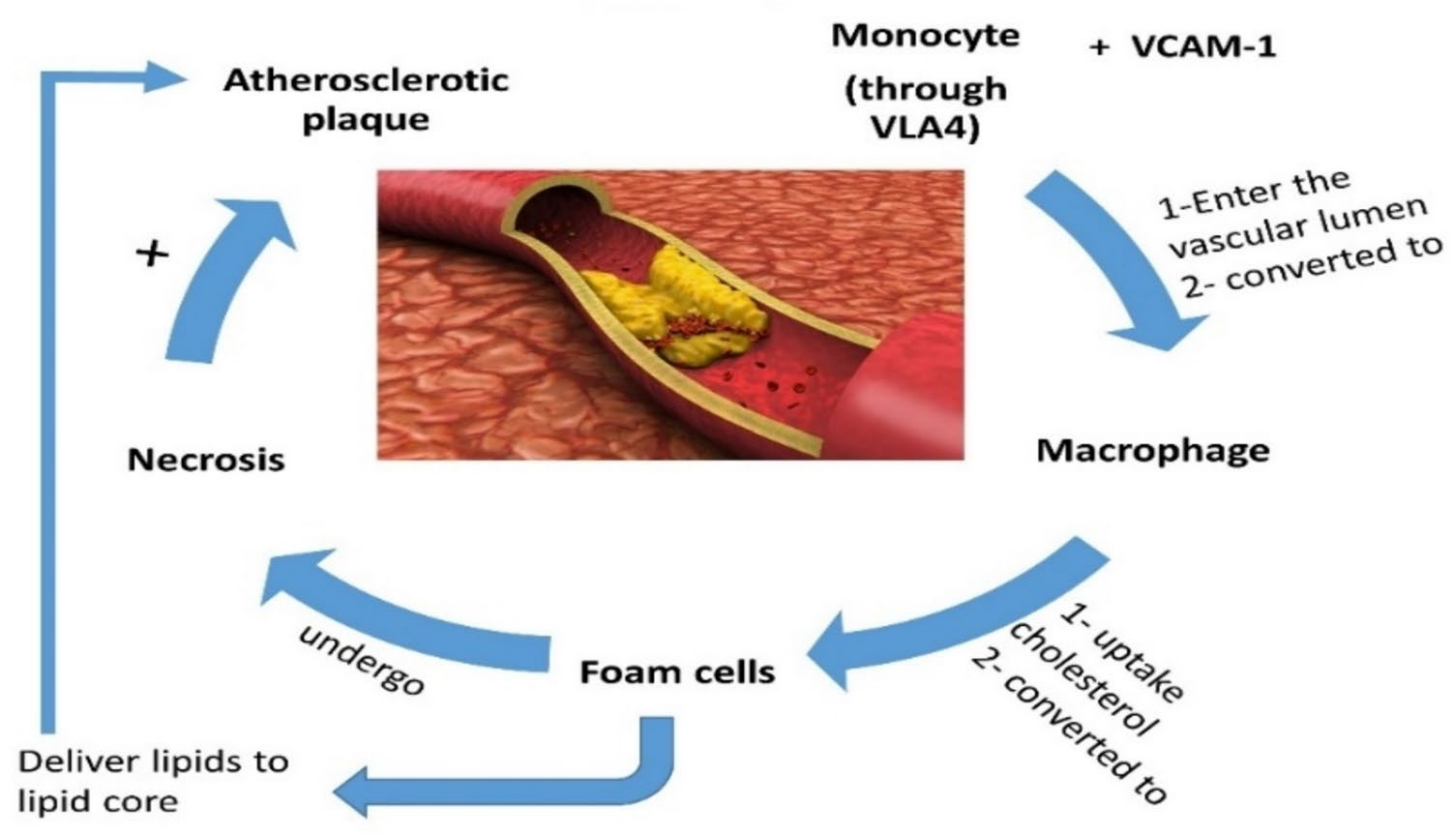
Pathophysiology of atherosclerosis: monocyte via very late antigen 4 (VLA4) binds vascular cell adhesion molecule 1 (VCAM-1) and enters the vascular lumen, then is converted to macrophage which uptakes cholesterol and converted to foam cells that undergo necrosis and deliver lipid into the lipid core with formation of atherosclerotic plaque

Particularly, macrophage is the most immune cell intricate with the progression of AS and atherosclerotic complications including erosion and rupture, as macrophages consume oxidized ox-LDL with production of reactive oxygen species (ROS) (Jinnouchi et al. 2020). Excessive production of ROS promotes the development of oxidative stress and progression of plague instability (Poznyak et al. 2020a, b). Consequently, ox-LDL accelerates macrophage oxidative stress process and oxidative stress which together with inflammation enhance AS progression in a vicious cycle as inflammation induces oxidative stress and vice versa. It has been revealed that ox-LDL activates infiltration of monocytes and migration of smooth muscle cells, it contributes to atherothrombosis via induction apoptosis of endothelial cells, plaque erosion, production of tissue factors, and impairment of endogenous anticoagulant pathway (Wang et al. 2019). In usual physiological condition, HDL attenuates production and effect of ox-LDL. In addition, oxidized HDL (ox-HDL), which is loss of its vasculoprotective effect, acts as pro-inflammatory and pro-atherogenic mediator, and increases risk of AS progression (He et al. 2018). Remarkably, ox-HDL can induce the progression of atherosclerotic plaque erosion and rupture. Hence, ox-HDL is regarded as a potential risk factor for AS and atherothrombosis (He et al. 2018). Oxidative stress stimulates the expression of inflammatory signaling pathway, pro-inflammatory cytokines, and chemokines which in turn enhance ROS generation (Lee et al. 2010). NADPH oxidase which is highly expressed in vascular smooth muscle is involved in ROS generation. Higher expression of NADPH oxidase is increased by aging process leading to endothelial dysfunction, vascular inflammation, mitochondrial dysfunction, and oxidative stress (Ho et al. 2022). These observations revealed that AS pathogenesis is a complex process linked with dyslipidemia and associated inflammatory disorders and oxidative stress (Fig. 2).

## Role of VPN in AS

### VPN and inflammation

AS is regarded as an inflammatory disease; atherosclerotic plaque acts as a pathogen-associated molecular pattern (PAMP) which provokes immune response and infiltration of inflammatory cells. Endothelial injury by ox-LDL promotes the expression of adhesion molecules that increase recruitment of monocytes with the release of pro-inflammatory cytokines (Ou et al. 2019). PAMP promotes activation of NF-*κ*B which is a master regulator of immune response leading to the expression and release of pro-inflammatory cytokines (Wu et al. 2019). Therefore, inhibition of NF-*κ*B prevents the expression of adhesion molecules, release of pro-inflammatory cytokines, and development of inflammatory reactions in AS. Experimental studies illustrated that inhibition of NF-κB signaling pathway abrogates the development of AS in mice (Mallavia et al. 2013). VPN has anti-inflammatory effect by inhibiting the expression of NF-*κ*B through stabilization of I*κ*B*α* (Wang et al. 2014a, b). Zhuang et al. showed that VPN inhibits AS in mice through suppression of NF-*κ*B pathway. Importantly, ox-LDL activates NF-*κ*B trough stimulation of IKK*α*/*β*, I*κ*B*α*, Akt, and PI3K/Akt signaling pathway. VPN inhibits IKK*α*/*β* and I*κ*B*α* preventing NF-*κ*B activation and inflammatory reactions by targeting of IKK*α*/*β* which is the main pathway for the anti-inflammatory effect of VPN. (Zhuang et al. 2013b). Interestingly, VPN inhibits NF-*κ*B-dependent inflammatory responses by targeting IKK. VPN constrains intracellular IKK kinase activation and NF-*κ*B-dependent transcriptional activity. VPN inhibits NF-*κ*B-dependent inflammatory responses by suppressing IKK, independent of PDE1 activity (Jeon et al. 2010).

Migration and transformation of monocytes to the foam ells are mediated by NF-*κ*B and monocyte chemoattractant protein 1 (MCP-1), MCP-1 promotes the expression of scavenger receptor and differentiation of monocytes to foam cells (Tabata et al. 2003). Accumulation of ox-LDL in the monocytes triggers differentiation of monocytes to macrophage and foam cells. MCP-1 increases CD36 expression which promote trans-endothelial migration of monocytes (Fujiwara and Kobayashi 2005). Different studies highlighted that NF-*κ*B increases MCP-1 expression in various inflammatory disorders. Therefore, inhibition of NF-*κ*B by VPN may reduce differentiation of monocytes to macrophage and formation of foam cells in vascular wall.

Inflammation is a key factor involved in all stages of AS progression. Cells that intricate in the pathogenesis of AS are activated by soluble factors which influence disease progression. Pro-inflammatory cytokines accelerate AS progression, while anti-inflammatory cytokines ameliorate AS (Fatkhullina et al. 2016). Certainly, cytokines are produced by and act (often synergistically) on almost all cells intricate in the pathogenesis of AS, contributing in all steps of the process, from the early endothelial dysfunction to the late formation and disruption of a vulnerable plaque (Tousoulis et al. 2016). Pro-atherogenic cytokines such as tumor necrosis factor-alpha (TNF-*α*), interleukin (IL)-1, and IL-6 are secreted by macrophages, lymphocytes, natural killer cells, and vascular smooth muscle cells. TNF-*α* and IL-1 signaling is mostly mediated by the p38 mitogen-activated protein kinase (p38MAPK)/NF-*κ*Β pathway, and this affects almost all cells involved in atherogenesis by promoting the expression of cytokines, adhesion molecules, and the migration and mitogenesis of vascular smooth muscle and endothelial cells (Tousoulis et al. 2016). Most of the pro-inflammatory cytokines released from macrophages are inhibited by the action of VPN via NF-*κ*B-dependent mechanism. VPN blocks monocyte adhesion and migration by inhibiting expression and action of MCP-1 (Akhter et al. 2021). Though VPN has selective inhibitory activity on the PDE1 enzyme, and its anti-inflammatory effects seem to be PDE1-independent. VPN inhibits LPS-induced lung inflammation in mice by targeting NF-*κ*B activation and, consequently, the production of NF-*κ*B-related cytokines TNF-α and IL-1*β*, as well as the recruitment of polymorph nuclear cells (Jeon et al. 2010). Thus, VPN exhibited anti-atherogenic effect through inhibition of NF-*κ*B activation and the production of inflammatory cytokines, ROS, and other inflammatory mediators in a PDE1-independent manner (Zhuang et al. 2013a). In a model of endotoxemia, intraperitoneal administration of VPN reduced hippocampal expression of IL-1*β* and TNF-*α* after intraperitoneal administration of LPS (Gómez et al. 2014). VPN reduces acetic acid-induced visceral nociception, validating the possible use of VPN in inflammatory pain conditions (Abdel Salam 2006). Nevertheless, it was not determined if similar analgesic and anti-inflammatory effects could be obtained by oral treatment with VPN in LPS-induced pain (Abdel Salam 2006). The protective effect of VPN against ischemic reperfusion injury (IRI) could be attributed to modulation of NADPH oxidase/Nrf2, IKK*β*/NF-*κ*B p65, and cleaved caspase-3 expressions. Thus, VPN could improve oxidant/antioxidant balance, suppress triggered inflammatory response, and promote renal cell survival after IRI (Azouz et al. 2022). These verdicts suggest that VPN may be effective against development of inflammatory disorders in AS.

### VPN and plaque stability

Most acute cardiovascular events are due to thrombotic occlusion caused by ruptured atherosclerotic plaques. Plaque rupture is mostly caused by an inflammatory degradation of the plaque connective tissue, most importantly the fibrous cap (Wang et al. 2019). Atherosclerotic plaques that are at high risk of rupturing are often referred to as vulnerable plaques. Such plaques are characterized by abundant inflammation, a large core of lipids and necrotic cells, and a thin fibrous cap. The plasma level of certain pro-inflammatory cytokines can be used as surrogate markers for the identification of patients with high-risk plaques (Wang et al. 2019). Thus, high cytokines within the atherosclerotic plaques may diffuse into the circulation, and thereby plasma levels of such cytokines could reflect the inflammatory activity in the plaques. Plasma levels of several cytokines have been shown to correlate with the progression of the AS or have been considered as markers of cardiovascular events (Wang et al. 2019).

It has been shown that macrophages within atherosclerotic plaque are regarded as the major source of pro-inflammatory and inflammatory cytokines, and macrophage is considered as a key regulator of metabolic signals and inflammatory response in atherosclerotic plaque formation. Therefore, macrophage activity and plaque contents change in a dynamic balance. Macrophage lipid contents trigger inflammation and immune response by augmentation of the sensitivity of TLR4 to their ligands by inducing the expression of nod-like receptor pyrin 3 (NLRP3) inflammasome (Moore et al. 2013). Of note, interaction of ox-LDL with monocytes/macrophages in the atherosclerotic plaque promotes inflammatory and oxidative stress disorders (Yuan et al. 2019).

VPN attenuates the formation of atherosclerotic lesion in ApoE knockout mice. Besides, in vitro study demonstrated that VPN blocks uptake of ox-LDL by cultured macrophage through inhibition of the expression of ox-LDL receptor (Cai et al. 2013). VPN has weak inhibitory effect on the formation of foam cells, though it has strong inhibitory effect on ROS generation and the expression of pro-inflammatory cytokines in macrophages of atherosclerotic plaque as it attenuates uptake of ox-LDL by foam cells (Zhuang et al. 2013b).

It has been shown that VPN suppresses carotid intimal hyperplasia through inhibition of vascular smooth muscle cells (VSMCs) migration and proliferation (Cai et al. 2012). Wang et al. showed that VPN reduces carotid neo-intimal hyperplasia in diabetic rat following balloon injury (Wang et al. 2014a, b). Migration and proliferation of VSMCs are regulated by platelet derived growth factor (PDGF) which secreted from endothelial cells, PDGF promotes VSMCs phenotype shift during vascular injury by regulating autophagy (Han et al. 2021). Likewise, migration and proliferation of VSMCs are also augmented by Akt and extracellular signal regulated protein kinase 1/2 (ERK1/2) but by different pathway (Yu et al. 2018). In vitro study demonstrated in two- and three-dimensional assay that VPN blocks PDGF and ERK1/2-mediated VSMCs (Cai et al. 2012). These observations suggest that VPN induces a protective effect against intimal hyperplasia and vascular remodeling during development of AS.

Moreover, VPN stabilizes atherosclerotic plaque by increasing collagen content, plaque cap thickness and decreasing lipid-rich core size through attenuation of the expression of MMP-9 and TNF-*α* (Zhuang et al. 2013b). MMP-9 induces degradation of plaque matrix causing plaque necrosis, rupture, and thrombosis. Inhibition of MMP-9 by VPN promotes plaque stability and prevents plaque-mediated complications.

Consequently, VPN may play a crucial role in preventing development of atherosclerotic complication via enhancement of plaque stability.

### VPN and PDE in AS

It has been shown that PDE-1A is highly expressed in foam cells of atherosclerotic plaque while PDE-1B and PDE-1C are highly abundant in VSMCs. Different studies illustrated that PDE inhibitors by increasing cAMP/cGMP in VSMCs can reduce intima media thickness and progression of AS (Priksz et al. 2018). A prospective, randomized study involved 329 T2DM patients treated with cilostazol (PDE-3 inhibitor) showed that cilostazol reduced intima media thickness compared to aspirin (Katakami et al. 2010). As well, sildenafil (PDE-5 inhibitor) improves endothelial dysfunction and vascular injury in mice (Priksz et al. 2018). As VPN is a selective PDE1 inhibitor, but it also increases cAMP/cGMP in VSMCs with subsequent inhibition of migration and differentiation. Intracellular cAMP is regulated by PDE and adenylyl cyclase that control atherogenesis via modulation recruitment and migration of monocytes as well as differentiation of macrophages to foam cells (Fantidis 2010). It has been shown that cAMP inhibits the release of pro-inflammatory cytokines from differentiated macrophage in the atherosclerotic plaque. Besides, cAMP attenuates progression of atherosclerotic plaque by reducing macrophage cholesterol content via expression of macrophage secretory pathway. In addition, increasing of cAMP is associated with inhibition expression of MMP-9, platelet activation, thrombosis, and atherogenesis (Zhou et al. 2017). Likewise, cGMP plays a critical role in maintaining endothelial function through induction of the release of NO. It has been observed that alteration of cGMP/NO signaling pathway is associated with atherogenesis and development of AS. Experimental study demonstrated that chronic hypercholesterolemia-induced ROS reduce availability of vasoprotective NO in rabbit. This effect was mediated by reducing cGMP which induce dysfunction of intima proliferation and endothelial through regulation of survival and phenotypic plasticity of VSMCs for the development of AS (Melichar et al. 2004). VSMCs express different types of cGMP effectors like cGMP-dependent protein kinase and NO-sensitive guanylyl cyclase which is involved in the inhibition of VSMCs proliferation. Therefore, cGMP has a major role in regulating atherogenesis by inhibiting VSMCs proliferation (Lehners et al. 2018). Thus, VPN by increasing cAMP/cGMP could be effective in the management of AS by maintaining endothelial integrity and inhibition of VSMCs proliferation (Zhang and Yan 2020).

### VPN, oxidative stress, and endothelial dysfunction in AS

Oxidative stress is intricate in the pathogenesis of various cardiovascular diseases including AS. AS represents state of oxidative stress characterized by protein and lipid oxidations in the vascular endothelium (Poznyak et al. 2020a, b). Overproduction of ROS is linked with the development and progression of endothelial dysfunction and AS (Jacinto et al. 2018). Notably, oxidative stress is an integral factor involved in the pathogenesis of AS that occurs in parallel with activations of chemokines and pro-inflammatory cytokines (Peluso et al. 2012). It is well-known that the major risk factors for AS such as cardiovascular diseases, dyslipidemia, and T2D are commonly associated with oxidative stress (Sinyov et al. 2017). Disturbance of the endothelial function is measured to be the first key event in atherogenesis progression, which distorts the balance between vasoconstriction and vasodilatation, increases the endothelial permeability, and activates a local inflammatory response. It results in the infiltration of inflammatory cells from circulation into the vessel wall and indirect induction of cytokines and other inflammatory mediators (Perrotta et al. 2015). A case–control study illustrated that biomarkers of oxidative stress such as MDA and monocyte superoxide anion were higher in patients with carotid AS. However, treatment with antioxidant *α*-tocopherol did not reduce carotid AS (Devaraj et al. 2007). A prospective study showed that oxidative stress index was high at baseline and reduced following appropriate management in patients with AS (Belce et al. 2022). Biomarkers of oxidative stress such as ox-LDL and ox-HDL were increased, whereas biomarkers of antioxidants such as catalase, arylesterase, and paraoxonase were reduced in patients with AS (Belce et al. 2022). Interestingly, statins therapy reduces the biomarkers of oxidative stress in patients with AS (Mayyas et al. 2018). These findings indicated that oxidative stress is involved in the pathogenesis of AS. However, the potential role of oxidative stress in the pathogenesis of AS is mainly related to induction of endothelial dysfunction.

Oxidative stress-induced endothelial dysfunction is mediated by depletion of endothelium NO (Al-Kuraishy et al. 2018). It has been illustrated that oxidative stress promotes the formation of atherosclerotic plaque through induction expression of adhesion molecules, inflammation, and development of endothelial dysfunction (Marchio et al. 2019). Endothelial NADPH oxidase is a master enzyme for generation of ROS that correlate with state of endothelial dysfunction and AS (Poznyak et al. 2020a, b). Different studies revealed that LDL directly activates endothelial NADPH oxidase by activating signal transductions such as phospholipase A2 and release of arachidonic acid (AA) which is intricate in activation of NADPH oxidase (Hussien et al. 2021; Manea et al. 2020). Monocytes, macrophages, VSMCs, and endothelial cells have ability to oxidize LDL through NADPH oxidase-dependent pathway (Lixia et al. 2021). Moreover, ROS are also produced by other enzymes and pathways including xanthine oxidase, mitochondrial eNOS, and uncoupling eNOS (Poznyak et al. 2020a, b). The vascular endothelium is protected from the effect of oxidative stress by antioxidant enzyme system including catalase, superoxide dismutase, paraoxonase, and glutathione peroxidase (Al-Thomali et al. 2022). ROS induces atherogenesis by oxidative modification of phospholipids and lipoproteins. Therefore, oxidative/antioxidant imbalance promotes macrophage polarization, formation of foam cells, and induction of atherosclerotic plaque (Khatana et al. 2020).

VPN is regarded as an antioxidant agent that reduces propagation of oxidative stress by inhibiting generation of ROS in different inflammatory and oxidative stress disorders (Al-Kuraishy, Al-Gareeb, and Al-Nami 2019; Ruiz-Miyazawa et al. 2015). In response to LPS, both macrophages and neutrophils through TLR4/NF-*κ*B produce large number of mediators including ROS and pro-inflammatory cytokines leading to inflammation and associated oxidative stress (Ruiz-Miyazawa et al. 2015). Experimental study revealed that administration of VPN 30 mg/kg inhibits LPS-induced oxidative stress in mice through inhibition of NF-*κ*B signaling. Al-kuraishy et al. revealed that VPN attenuates gentamicin-induced acute kidney injury in rats by inhibiting oxidative stress (Al-Kuraishy, Al-Gareeb, and Al-Nami 2019). Recently, VPN-induced inhibition of PDE1 prevents brain oxidative stress in behavioral phenotype of autism spectrum disorders (Luhach et al. 2021). Additionally, VPN mitigates inflammatory and oxidative stress disorders in COVID-19 which is linked with hyperinflammation and oxidative stress (Al-Kuraishy et al. 2022b). These findings proposed that VPN could be effective in reducing oxidative stress, a cornerstone in the pathogenesis of AS, through inhibition of NF-*κ*B and PDE1.

**Fig. 3:**
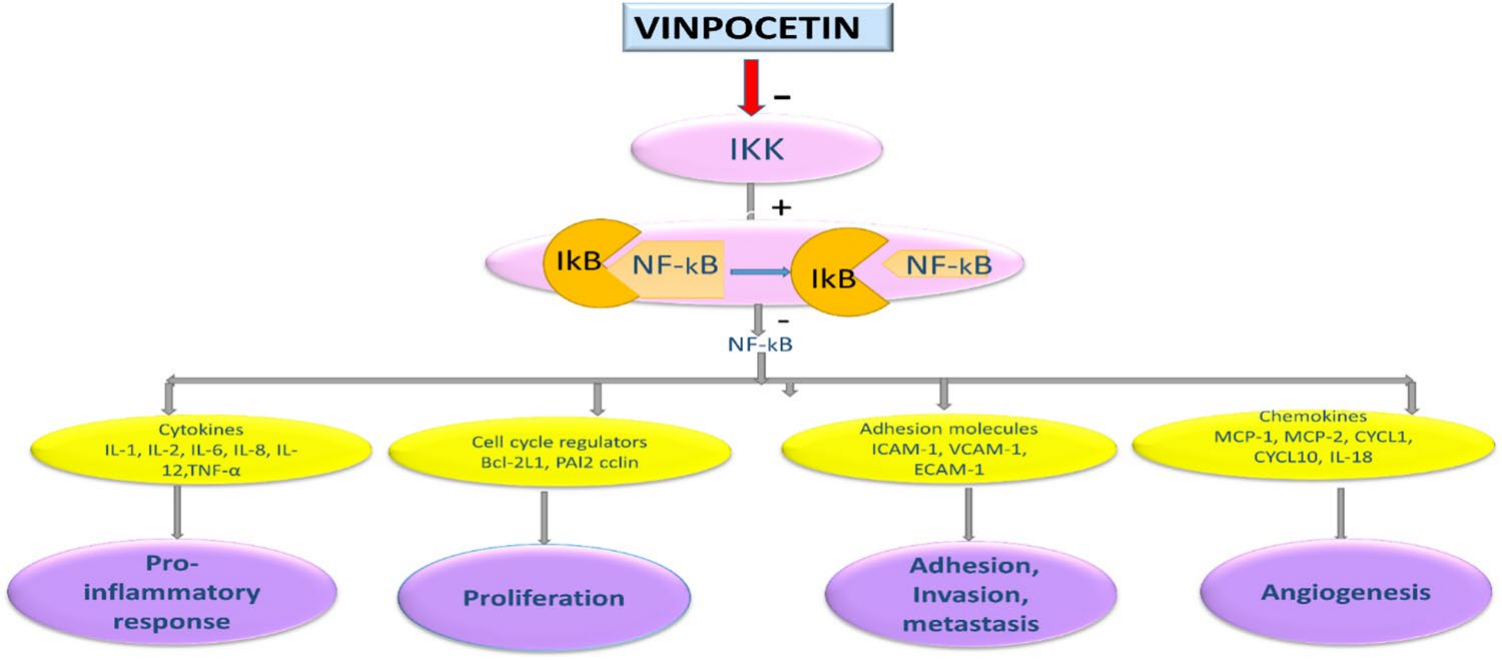
Role of vinpocetine in atherosclerosis: vinpocetine inhibits NF-κB and related signaling leading to suppression release of pro-inflammatory cytokines, reactive oxygen species (ROS), proliferation, and migration of vascular smooth muscle cells

Furthermore, hemodynamic shear stress is a frictional force on the vascular endothelium that controls endothelium homeostasis in normal physiological process, the laminar shear stress prevents endothelial injury and development of AS whereas disturbed blood flow induces atherothrombosis (Huynh and Heo 2019). It has been reported that disturbed blood flow due to endothelial dysfunction in AS induces activation of endothelial voltage-gated Na^+2^ channel which promotes the expression of ERK1/2 and NF-*κ*B activation (Gilbert et al. 2017). VPN is regarded as a potent inhibitor of voltage-gated Na^+2^ channel preventing cell toxicity and death through mitigation of activated ERK1/2 and NF-*κ*B (Bönöczk et al. 2000). Taken together, VPN through mitigation of inflammatory and oxidative stress with plaque stability effects could be effective agent in the management of AS (Fig. 3).

## Conclusion

VPN is used in the management of different cerebrovascular disorders. VPN is a potent inhibitor of PDE1, and has anti-inflammatory and antioxidant effects through inhibition of the expression of NF-*κ*B. VPN has been shown to be effective against development and progression of AS. Most of the pro-inflammatory cytokines released from macrophages are inhibited by action of VPN via NF-*κ*B-dependent mechanism. Importunately, ox-LDL activates NF-*κ*B by triggering of IKK*α*/*β*, I*κ*B*α*, Akt, and PI3K/Akt signaling pathway. VPN prevents NF-*κ*B activation and inflammatory reactions. Targeting of IKK*α*/*β* is the main pathway for the anti-inflammatory effect of VPN. Furthermore, VPN blocks monocyte migration by inhibiting the release of pro-inflammatory cytokines. Accumulation of ox-LDL in the monocytes triggers differentiation of monocytes to macrophage and foam cells. MCP-1 increases the expression of CD36 which promotes trans-endothelial migration of monocytes. NF-*κ*B increases expression of MCP-1 in various inflammatory disorders. Therefore, inhibition of NF-*κ*B by VPN may reduce differentiation of monocytes to macrophage and foam cells. Remarkably, most of the pro-inflammatory cytokines released from macrophages are inhibited by action of VPN via NF-*κ*B-dependent mechanism. In addition, VPN may play a crucial role in preventing development of atherosclerotic complication by improving plaque stability. Besides, VPN through increasing of cAMP/cGMP could be effective in the management of AS by maintaining endothelial integrity and inhibition of VSMCs proliferation. VPN is effective in reducing oxidative stress, a cornerstone in the pathogenesis of AS, through inhibition of NF-*κ*B and PDE1.

Taken together, VPN promotes plaque stability and prevents erosion and rupture of atherosclerotic plaque. Though, this review cannot give the final conclusion regarding the atheroprotective role of VPN in AS. Herein, preclinical and clinical studies are reasonable in this regard.

## Data Availability

All data generated or analyzed during this study are included in this published article.
